# Correction: Determinants of domestic violence against women in Cambodia: How digital access, media exposure, motorcycle ownership, and partners’ alcohol use matter

**DOI:** 10.1371/journal.pone.0362222

**Published:** 2026-11-03

**Authors:** Samnang Um, Sopheap Suong, Chantrea Sieng, Sovandara Heng, Grace Marie Ku, Sothy Heng

The last two authors, Grace Marie Ku and Sothy Heng should be noted as joint senior authors of this work.

The fifth author’s initials appear incorrectly in the citation. The correct citation is: Um S, Suong S, Sieng C, Heng S, Ku GM, Heng S (2026) Determinants of domestic violence against women in Cambodia: How digital access, media exposure, motorcycle ownership, and partners’ alcohol use matter. PLoS One 21(3): e0329981. https://doi.org/10.1371/journal.pone.0329981.
