# Supplementary material for: Determinants of domestic violence against women in Cambodia: How digital access, media exposure, motorcycle ownership, and partners’ alcohol use matter
Source: PLoS One. 2026 Mar 23;21(3):e0329981. doi: 10.1371/journal.pone.0329981 (PMC13008099; doi:10.1371/journal.pone.0329981)
Supplement: S1 Table — (DOCX) [file pone.0329981.s001.docx]

**S1 Table.** Variance Inflation Factor (VIF) for Sexual Violence Model

| **Predictor Variable** | **VIF** | **1/VIF** |
| --- | --- | --- |
| Women's Age | 2.0 | 0.491 |
| Partner's Age | 2.0 | 0.499 |
| Internet use | 1.7 | 0.581 |
| Mobile phone ownership | 1.7 | 0.587 |
| Wealth index | 1.6 | 0.627 |
| Women's education | 1.5 | 0.659 |
| Partner’s education | 1.3 | 0.747 |
| Residence (urban/rural) | 1.2 | 0.813 |
| Media exposure | 1.1 | 0.941 |
| Motorcycle Ownership | 1.1 | 0.953 |
| Partner's Alcohol Use | 1.0 | 0.994 |
| **Mean VIF** | **1.48** |  |
