# Supplementary material for: Determinants of domestic violence against women in Cambodia: How digital access, media exposure, motorcycle ownership, and partners’ alcohol use matter
Source: PLoS One. 2026 Mar 23;21(3):e0329981. doi: 10.1371/journal.pone.0329981 (PMC13008099; doi:10.1371/journal.pone.0329981)
Supplement: Attachment — Submitted filename: Recommendation.docx [file pone.0329981.s006.docx]

1. Recommendation (Answer options: Accept, Minor Revision, Major Revision, Reject) - Minor revision
2. Is the manuscript technically sound, and do the data support the conclusions? (Answer options: Yes, No, Partly) – Yes
3. Has the statistical analysis been performed appropriately and rigorously? (Answer options: Yes, No, I don’t know, N/A) Yes
4. Does the manuscript adhere to the PLOS Data Policy? Additional details can be found at http://journals.plos.org/plosone/s/materials-and-software-sharing. (Answer options: Yes, No) Yes
5. Is the manuscript presented in an intelligible fashion and written in standard English? (Answer options: Yes, No) Yes
6. Review Comments to the Author (minimum 200 characters): The authors made significant contribution to discourse on domestic violence. While the scholarship was cohesive and comprehensible, the authors did not clearly project policy implication of the study. I could deduce programmatic outcomes, but policies make these interventions more sustainable.
7. PLOS authors have the option to publish the peer review history of their article (https://journals.plos.org/plosone/s/editorial-and-peer-review-process#loc-peer-review-history). If published, this will include your full peer review and any attached files. Do you want your identity to be public for this peer review? (Answer options: Yes, No) No
   7a. If you answered "Yes" above, that you would like your identity to be public, please provide your full name, as it should appear on the published peer review. Please do not sign the review on behalf of another person.

   Confidential to Editor:
1. Do you have any potentially competing interests? If none, type "None." Our policy on competing interests can be found at <http://journals.plos.org/plosone/s/competing-interests>. No
2. Did you receive any assistance in preparing this review (e.g. from a post-doc or graduate student)? If yes, please provide their name and title. If you have chosen to reveal your identity to the authors in question 7 above, please include the name of the person who assisted you in the response to question 7 as well. (optional) No
3. Do you want to receive recognition for this review on a Web of Science researcher profile? (https://clarivate.com/products/scientific-and-academic-research/research-discovery-and-workflow-solutions/researcher-profiles/)? If you opt in, your Web of Science profile will automatically be updated to show a verified record of this review in full compliance with the journal's review policy. If you don't have a Web of Science profile, you will need to first create a free account. (Answer options: Yes, No) Yes
