# Supplementary material for: Determinants of domestic violence against women in Cambodia: How digital access, media exposure, motorcycle ownership, and partners’ alcohol use matter
Source: PLoS One. 2026 Mar 23;21(3):e0329981. doi: 10.1371/journal.pone.0329981 (PMC13008099; doi:10.1371/journal.pone.0329981)
Supplement: Attachment — Submitted filename: My comments.docx [file pone.0329981.s007.docx]

**Reviewer’s comments**

The article examined how digital access, media exposure, motorcycle ownership and partners' alcohol use are associated with violence types: physical, sexual, emotional, and intimate partner violence (IPV), while controlling for socio-demographic factors. This is timely and relevant perhaps with the rising cases of intimate partner violence in our contemporary society.

However, the authors need to clarify ‘violence types’ and ‘intimate partner violence (IPV)’. The way they are being currently used in the article are confusing or similar: violence types (physical, sexual and emotional) and intimate partner violence. There is the need for further clarifications.

Also, the authors have not provided clear and convincing evidence on the novelty of this study in Cambodia. I have seen a number of empirical studies on how digital access, media exposure and partners' alcohol affect intimate partner violence (IPV). The only novelty I could agree with is ‘motorcycle ownership’.

Also, the article lacks literature review where gaps ought to have emerged. Due to lack of literature, there are no conceptual, theoretical and empirical debates and engagements. Furthermore, the study does not have clear theoretical framework.

While methodology section contains important elements, it lacks justifications. The authors have not really justified most of their methodological choices. The article will benefit from proper and adequate justifications of each methodological choice made.

Generally, the analysis is good. Nonetheless, the authors need to improve their interpretations of key statistical results. In other words, the interpretations of key figures should be clear and explicit.

In addition, the authors need to improve on discussion of findings. For instance, novel findings should be incorporated with proper interpretations and justifications. This could be linked to dearth of conceptual, empirical and theoretical review. Also, implications to policies, practices and theories should be more explicit.

Overall, the article should be accepted for publication after the above issues have been attended to.
