# Supplementary material for: Determinants of domestic violence against women in Cambodia: How digital access, media exposure, motorcycle ownership, and partners’ alcohol use matter
Source: PLoS One. 2026 Mar 23;21(3):e0329981. doi: 10.1371/journal.pone.0329981 (PMC13008099; doi:10.1371/journal.pone.0329981)
Supplement: Attachment — Submitted filename: Response to Reviewers.docx [file pone.0329981.s009.docx]

**Response to Reviewers**

**Manuscript ID:** PONE-D-25-40112 **Title:** Determinants of Domestic Violence Against Women in Cambodia: How Digital Access, Media Exposure, Motorcycle Ownership, and Partners' Alcohol Use Matter

**Journal:** PLOS ONE

**Academic Editor:** Lanre Abdul-Rasheed Sulaiman, PhD

**Dear Dr. Sulaiman and Reviewers,**

Thank you for the insightful feedback on our manuscript. We appreciate your positive assessment of the study's merit and timely relevance. We have undertaken a major revision, addressing every point raised by the Academic Editor and the Reviewers. We believe these revisions significantly strengthen the manuscript's theoretical grounding, conceptual clarity, interpretation of findings, and policy relevance.

Below is a point-by-point response detailing the revisions made.

1. **Academic Editor Comments**

| **Editor Comment** | **Author Response** | **Location in Manuscript** |
| --- | --- | --- |
| Presentation of Terms ("violence types: physical, sexual, emotional, and intimate partner violence (IPV)") leads to ambiguity because the text often lists IPV as if it were a distinct form of domestic violence alongside the three specific forms of violence. | We agree that the simultaneous listing created ambiguity. We have comprehensively revised the terminology throughout the manuscript (Abstract, Introduction, Methods, Results, and Tables) to clarify that Intimate Partner Violence (IPV) is the overarching, composite term defined as the experience of any of the three specific forms: physical violence, sexual violence, or emotional violence perpetrated by an intimate partner. We ensure that IPV is no longer listed as a fourth, separate category. | Abstract, Introduction (para 1 & 2), Methods (Outcome Variable), Results (Table 2, Table 3, and text). |
| The manuscript does not explicitly name or detail a formal theoretical framework... | We acknowledge this critical omission. To provide a robust conceptual foundation, we have formally adopted the Ecological Model of Violence Prevention as our guiding framework. We have introduced a new, dedicated section in the Introduction to detail this model and explicitly link our independent variables (Digital Access, Media Exposure, Motorcycle Ownership, and Alcohol Use) to the relevant layers of the ecological framework (Individual, Relational, Community, and Societal). | NEW section in the Introduction: "**Theoretical Framework**." |
| The discussion of these null findings [motorcycle ownership and media exposure] attributes the lack of association to the possibility that 'mere access or exposure... may not be sufficient...' The authors do not extensively debate why motorcycle ownership... might fail to demonstrate a protective effect in Cambodia... | We have significantly expanded the Discussion section to provide a more rigorous debate and contextual interpretation of the null findings for motorcycle ownership and media exposure. In the Cambodian context, physical mobility (motorcycle ownership) alone may be insufficient to confer empowerment if not accompanied by a shift in restrictive gender norms or control over the asset. Similarly, we debate that media exposure needs to be coupled with critical media literacy to challenge patriarchal norms and translate into a protective effect. | Discussion section (new paragraphs dedicated to these two null findings). |
| The manuscript's policy suggestions... are often phrased as broad programmatic needs rather than specific policy mechanisms. | We have refined the policy suggestions to be more explicit, actionable, and focused on policy mechanisms rather than broad programmatic outcomes. We detail concrete suggestions like advocating for a National Strategy for Secure Digital Inclusion and mandating gender-sensitive digital literacy integration into national curricula, providing the policy layer needed to sustain programmatic efforts. | Abstract, Conclusion section (significantly revised). |

2. **Reviewer 1 Comments**

| **Reviewer 1 Comment** | **Author Response** | **Location in Manuscript** |
| --- | --- | --- |
| The authors did not clearly project the policy implications of the study. I could deduce programmatic outcomes, but policies make these interventions more sustainable. | We concur with this assessment. As noted in the response to the Editor, we have extensively revised the abstract and the Conclusion/Policy Implications section to articulate specific, high-level policy mechanisms (e.g., national strategies, mandated funding) that would underpin and sustain the suggested programmatic interventions (e.g., safe digital access, alcohol misuse prevention). | Abstract, Conclusion section (significantly revised). |

3. **Reviewer 2 Comments**

| **Reviewer 2 Comment** | **Author Response** | **Location in Manuscript** |
| --- | --- | --- |
| The authors need to clarify ‘violence types’ and ‘intimate partner violence (IPV)’. | This was fully addressed in response to the Editor (Section 1), ensuring that IPV is consistently presented as the composite term for the three specific forms of violence. | Abstract, Introduction, Methods (Outcome Variable), Results. |
| The authors have not provided clear and convincing evidence on the novelty of this study in Cambodia. | We have strengthened the Introduction by explicitly stating the study's novelty. We emphasize that while individual factors have been studied, the simultaneous analysis of digital access (dual role), media exposure, motorcycle ownership (mobility/asset control), and partner alcohol use within the specific context of the 2021-2022 Cambodian national data represents a novel, multi-sectoral approach critical for informing the national strategy. | Introduction (final paragraph). |
| Also, the article lacks literature review where gaps ought to have emerged. Due to lack of literature, there are no conceptual, theoretical and empirical debates and engagements. | We have significantly expanded the Introduction to include a more robust literature review and scholarly debate. This now includes: 1) A formalized Theoretical Framework (Ecological Model), and 2) Richer empirical engagement with literature surrounding the contradictory effects of digital access, the role of media in social norm change, and the context-specific nature of mobility/asset ownership (motorcycle) as empowerment tools. | Introduction (expanded paragraphs on Digital Access, Media Exposure, and Motorcycle Ownership), Introduction |
| The study does not have clear theoretical framework. | This has been addressed by incorporating the Ecological Model of Violence Prevention into a dedicated section (as noted above). | Introduction |
| ...it lacks justifications. The authors have not really justified most of their methodological choices. | We have improved the Methods section by adding explicit justifications for key choices, including: 1) The use of the 2021-2022 CDHS data as the most recent nationally representative survey; 2) The necessity of using the survey package in STATA to account for the complex sampling design and weights; and 3) The justification for the selection of variables into the adjusted multivariable logistic regression models. | Methods section (Data Source, Statistical Analysis). |
| Generally, the analysis is good. Nonetheless, the authors need to improve their interpretations of key statistical results. | We have revised the Results section to ensure that the interpretations of the Adjusted Odds Ratios (AORs) for all significant findings (e.g., smartphone ownership, low-frequency internet use, partner alcohol use, and wealth) are clear, explicit, and immediately follow the presentation of the relevant figures or tables. | Results section (Adjusted Odds Ratio narrative). |
| In addition, the authors need to improve on discussion of findings... Also, implications to policies, practices and theories should be more explicit. | We have significantly expanded the Discussion section to provide deeper, contextualized interpretations, particularly for the dual role of digital access and the null findings (as per the Editor's request). The Conclusion section has been revised to clearly state the study's implications for Theory (Ecological Model), Policy (specific mechanisms), and Practice (programmatic action). | Discussion section, Conclusion section (fully revised). |

4. **PLOS ONE Journal Requirements**

**We have noted the general journal requirements and will ensure the following actions are taken upon resubmission:**

| Requirement | Action Taken |
| --- | --- |
| 1. PLOS ONE's style requirements | We have reformatted the manuscript to strictly adhere to PLOS ONE’s style and formatting guidelines (file naming, citations, and structure). |
| 2. Code Sharing | This study used publicly available data from the Cambodia Demographic and Health Survey (CDHS) 2021–2022. The dataset is accessible through The DHS Program (<https://dhsprogram.com/data/available-datasets.cfm>) upon registration and approval. Analysis codes are available from the corresponding author upon reasonable request through email: [umsamnang56@gmail.com](mailto:umsamnang56@gmail.com) |
| 3. Data Availability Statement | We are revising the Data Availability Statement in the submission form. The data used is from the publicly available 2021-2022 CDHS. We will provide the exact source and URL in the Data Availability Statement and manuscript, explaining that access is available upon online registration with The DHS Program, thereby satisfying the open data policy. |
| 4. Data sharing plan before acceptance | As stated above, the data is from a public repository (DHS Program). We will clarify the public access path now, well before acceptance. |

We have uploaded the revised files: 1) ‘Response to Reviewers’, 2) ‘Revised Manuscript with Track Changes,’ and 3) ‘Manuscript’ (clean version). We thank you again for the opportunity to strengthen our manuscript and look forward to hearing from you.

Sincerely,

Samnang Um, PhD

On behalf of all co-authors
